# The genome sequence of the Fulvous Clothes Moth, *Tinea semifulvella* (Haworth, 1828)

**DOI:** 10.12688/wellcomeopenres.19079.1

**Published:** 2023-02-24

**Authors:** Douglas Boyes, University of Oxford and Wytham Woods Genome Acquisition Lab, Darwin Tree of Life Barcoding collective, Wellcome Sanger Institute Tree of Life programme, Wellcome Sanger Institute Scientific Operations: DNA Pipelines collective, Tree of Life Core Informatics collective, Clare Boyes, Darwin Tree of Life Consortium

**Affiliations:** 1 UK Centre for Ecology and Hydrology, Wallingford, UK; 2 Independent researcher, Wytham, UK

**Keywords:** Tinea semifulvella, Fulvous Clothes Moth, genome sequence, chromosomal, Lepidoptera

## Abstract

We present a genome assembly from an individual male *Tinea semifulvella* (the Fulvous Clothes Moth; Arthropoda; Insecta; Lepidoptera; Tineidae). The genome sequence is 596.6 megabases in span. The whole assembly is scaffolded into 45 chromosomal pseudomolecules, with the Z sex chromosome assembled. The mitochondrial genome has also been assembled and is 16.8 kilobases in length. Gene annotation of this assembly on Ensembl has identified 11,516 protein coding genes.

## Species taxonomy

Eukaryota; Metazoa; Ecdysozoa; Arthropoda; Hexapoda; Insecta; Pterygota; Neoptera; Endopterygota; Lepidoptera; Glossata; Ditrysia; Tineoidea; Tineidae; Tineinae; *Tinea*; *Tinea semifulvella* (Haworth, 1828) (NCBI:txid1101063).

## Background

T *inea semifulvella* is a micro-moth in the family Tineidae, a cosmopolitan group of moths, many associated with human habitation, and some of which have become pests. Although small (forewing length 6–10 mm), *T. semifulvella*, unlike many moths in the genus, is distinctive. It has a reddish head, and a dirty white forewing with the final third of the wing orangey-brown. There is a small dark dot on the back.

The moth is common and widespread throughout Britain, but more local in its distribution in Ireland. It occurs throughout Europe, and as far east as Iran ( Gaedike, 2019). It is on the wing between May and October and may well be double-brooded in the southern part of its UK range ( Sterling & Parsons, 2018). The moth is found in a range of habitats and comes to light. It is associated with bird’s nests, particularly those which occur in the open. This is unusual as most other moths found in bird nests have an association with hole-nesting species ( Boyes & Lewis, 2019). It has been suggested that this might be a strategy to avoid intraspecific competition ( Boyes, 2018). The moth has also been found on wool out of doors, dead animals ( Sterling & Parsons, 2018); and in hen-houses ( Gaedike, 2019), suggesting it is keratinophagous.

The genome of *T. semifulvella* was sequenced as part of the Darwin Tree of Life Project, a collaborative effort to sequence all named eukaryotic species in the Atlantic Archipelago of Britain and Ireland. Here we present a chromosomally complete genome sequence for *Tinea semifulvella* based on one male specimen from Wytham Woods, Oxfordshire, UK.

### Genome sequence report

**Figure 1.**
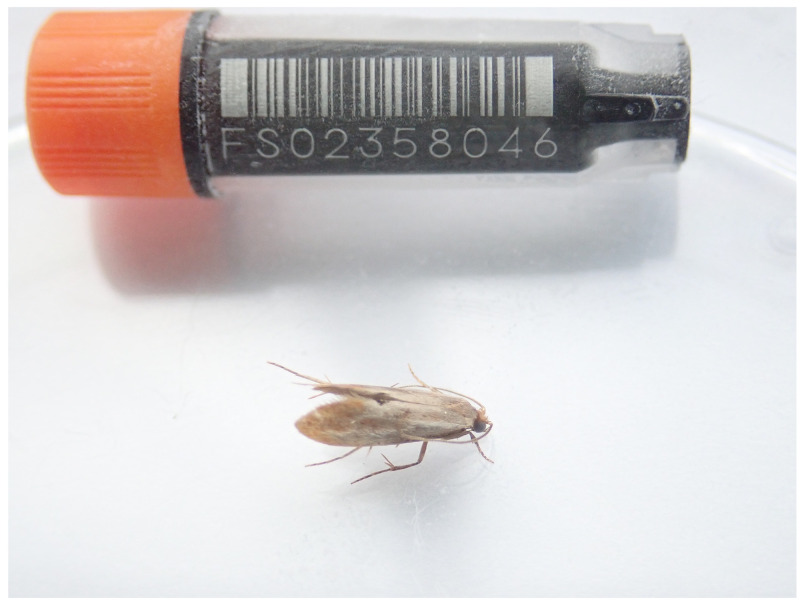
Photograph of the *Tinea semifulvella* (ilTinSemi1) specimen used for genome sequencing.

The genome was sequenced from one male *T. semifulvea* specimen ( Figure 1) collected from a grassland area of Wytham Woods (latitude 51.78, longitude –1.32). A total of 45-fold coverage in Pacific Biosciences single-molecule HiFi long reads and 62-fold coverage in 10X Genomics read clouds were generated. Primary assembly contigs were scaffolded with chromosome conformation Hi-C data. Manual assembly curation corrected 21 missing or mis-joins and removed one haplotypic duplication, reducing the scaffold number by 30.77% and increasing the scaffold N50 by 5.66%.

**Figure 2.**
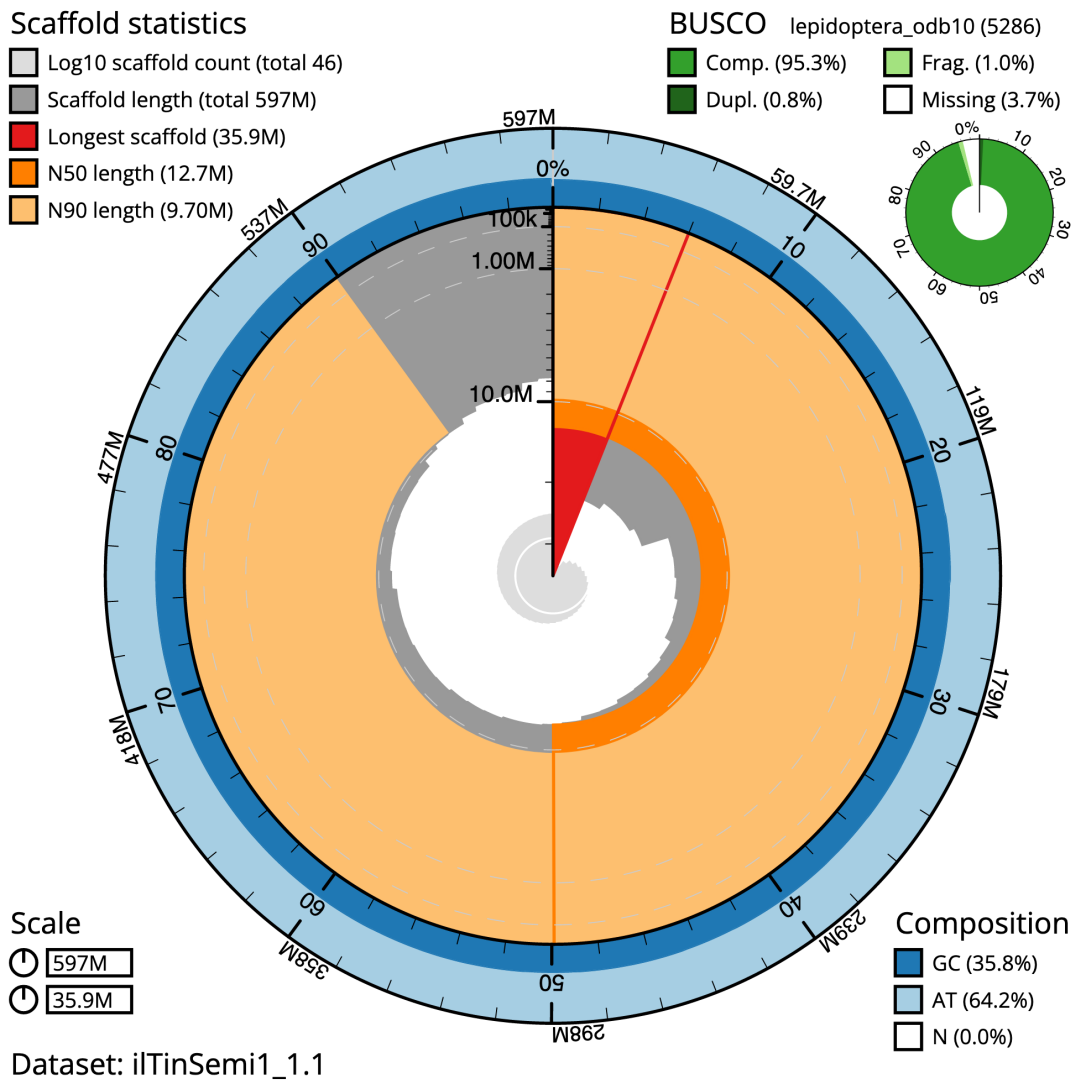
Genome assembly of *Tinea semifulvella*, ilTinSemi1.1: metrics. The BlobToolKit Snailplot shows N50 metrics and BUSCO gene completeness. The main plot is divided into 1,000 size-ordered bins around the circumference with each bin representing 0.1% of the 596,601,316 bp assembly. The distribution of scaffold lengths is shown in dark grey with the plot radius scaled to the longest scaffold present in the assembly (35,936,759 bp, shown in red). Orange and pale-orange arcs show the N50 and N90 scaffold lengths (12,715,305 and 9,702,360 bp), respectively. The pale grey spiral shows the cumulative scaffold count on a log scale with white scale lines showing successive orders of magnitude. The blue and pale-blue area around the outside of the plot shows the distribution of GC, AT and N percentages in the same bins as the inner plot. A summary of complete, fragmented, duplicated and missing BUSCO genes in the lepidoptera_odb10 set is shown in the top right. An interactive version of this figure is available at https://blobtoolkit.genomehubs.org/view/ilTinSemi1_1.1/dataset/ilTinSemi1_1.1/snail.

The final assembly has a total length of 596.6 Mb in 45 sequence scaffolds with a scaffold N50 of 12715305 Mb ( Table 1). All of the assembly sequence was assigned to 45 chromosomal-level scaffolds, representing 44 autosomes and the Z sex chromosome. Chromosome-scale scaffolds confirmed by the Hi-C data are named in order of size ( Figure 2– Figure 5; Table 2). The assembly has a BUSCO v5.3.2 ( Manni *et al.*, 2021) completeness of 95.3% (single 94.5%, duplicated 0.8%) using the lepidoptera_odb10 reference set. While not fully phased, the assembly deposited is of one haplotype. Contigs corresponding to the second haplotype have also been deposited.

**Table 1.** Genome data for *Tinea semifulvella*, ilTinSemi1.1.

Project accession data		
Assembly identifier	TinSemi1.1	
Species	*Tinea semifulvella*	
Specimen	ilTinSemi1	
NCBI taxonomy ID	1101063	
BioProject	PRJEB45131	
BioSample ID	SAMEA7520371	
Isolate information	ilTinSemi1 (PacBio and Chromium) ilTinSemi2 (RNASeq), ilTinSemi3 (Hi-C)	
Assembly metrics *		*Benchmark*
Consensus quality (QV)	58.1	*≥ 50*
*k*-mer completeness	99.99%	*≥ 95%*
BUSCO **	C:95.3%[S:94.5%,D:0.8%], F:1.0%,M:3.7%,n:5,286	*C ≥ 95%*
Percentage of assembly mapped to chromosomes	100%	*≥ 95%*
Sex chromosomes	Z chromosome	*localised homologous pairs*
Organelles	Mitochondrial genome assembled	*complete single alleles*
Raw data accessions		
PacificBiosciences SEQUEL II	ERR6608656	
10X Genomics Illumina	ERR6054822–ERR6054825	
Hi-C Illumina	ERR6054826	
PolyA RNA-Seq Illumina	ERR6363266	
Genome assembly		
Assembly accession	GCA_910589645.1	
*Accession of alternate haplotype*	GCA_910589255.1	
Span (Mb)	596.6	
Number of contigs	71	
Contig N50 length (Mb)	12.0	
Number of scaffolds	45	
Scaffold N50 length (Mb)	12.7	
Longest scaffold (Mb)	35.9	
Genome annotation		
Number of protein-coding genes	11,516	
Number of non-coding genes	2,198	
Number of transcripts	20,468	

* Assembly metric benchmarks are adapted from column VGP-2020 of “Table 1: Proposed standards and metrics for defining genome assembly quality” from ( Rhie *et al.*, 2021). ** BUSCO scores based on the lepidoptera_odb10 BUSCO set using v5.3.2. C = complete [S = single copy, D = duplicated], F = fragmented, M = missing, n = number of orthologues in comparison. A full set of BUSCO scores is available at https://blobtoolkit.genomehubs.org/view/ilTinSemi1_1.1/dataset/ilTinSemi1_1.1/busco.

**Figure 3.**
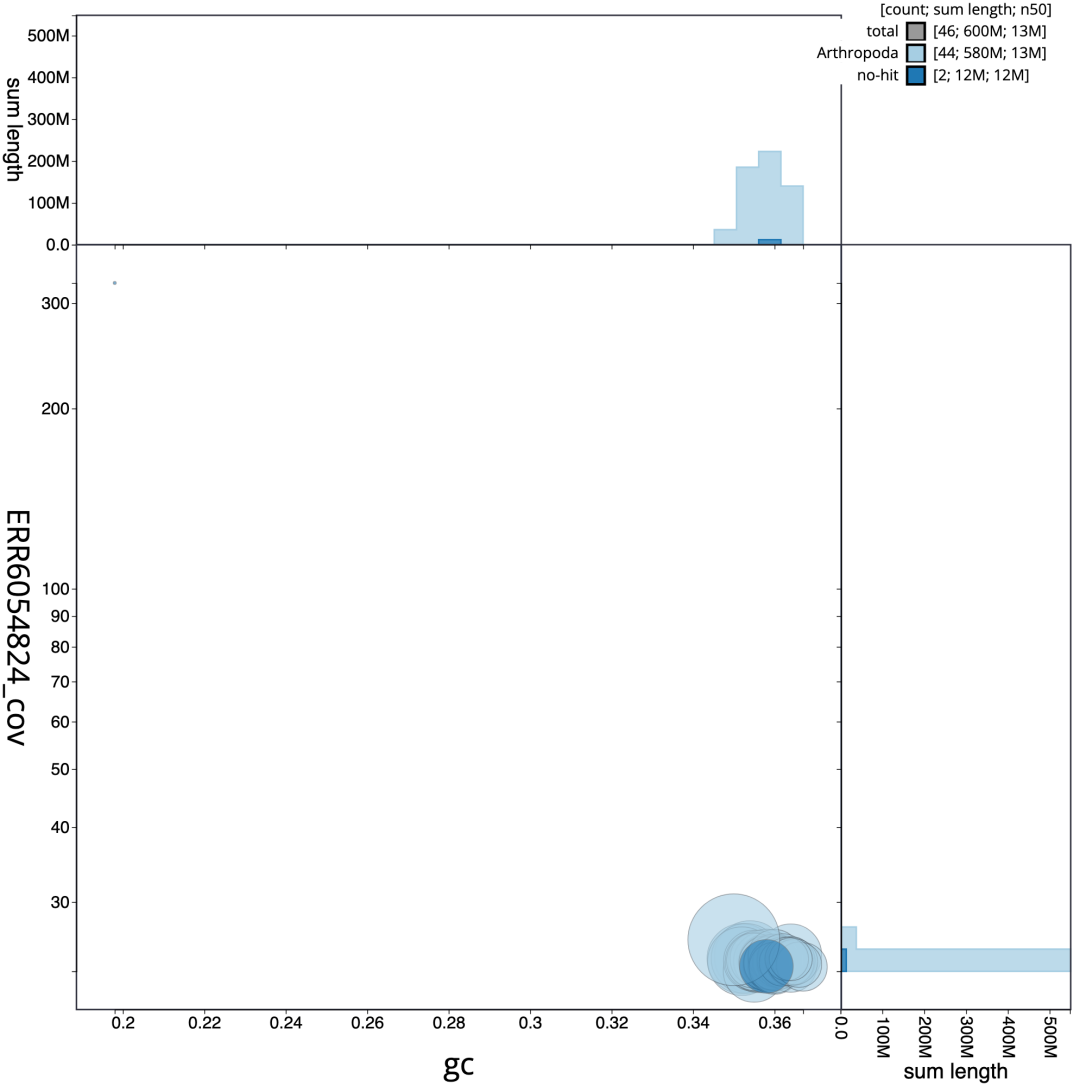
Genome assembly of *Tinea semifulvella*, ilTinSemi1.1: GC coverage. BlobToolKit GC-coverage plot. Scaffolds are coloured by phylum. Circles are sized in proportion to scaffold length. Histograms show the distribution of scaffold length sum along each axis. An interactive version of this figure is available at https://blobtoolkit.genomehubs.org/view/ilTinSemi1_1.1/dataset/ilTinSemi1_1.1/blob.

**Figure 4.**
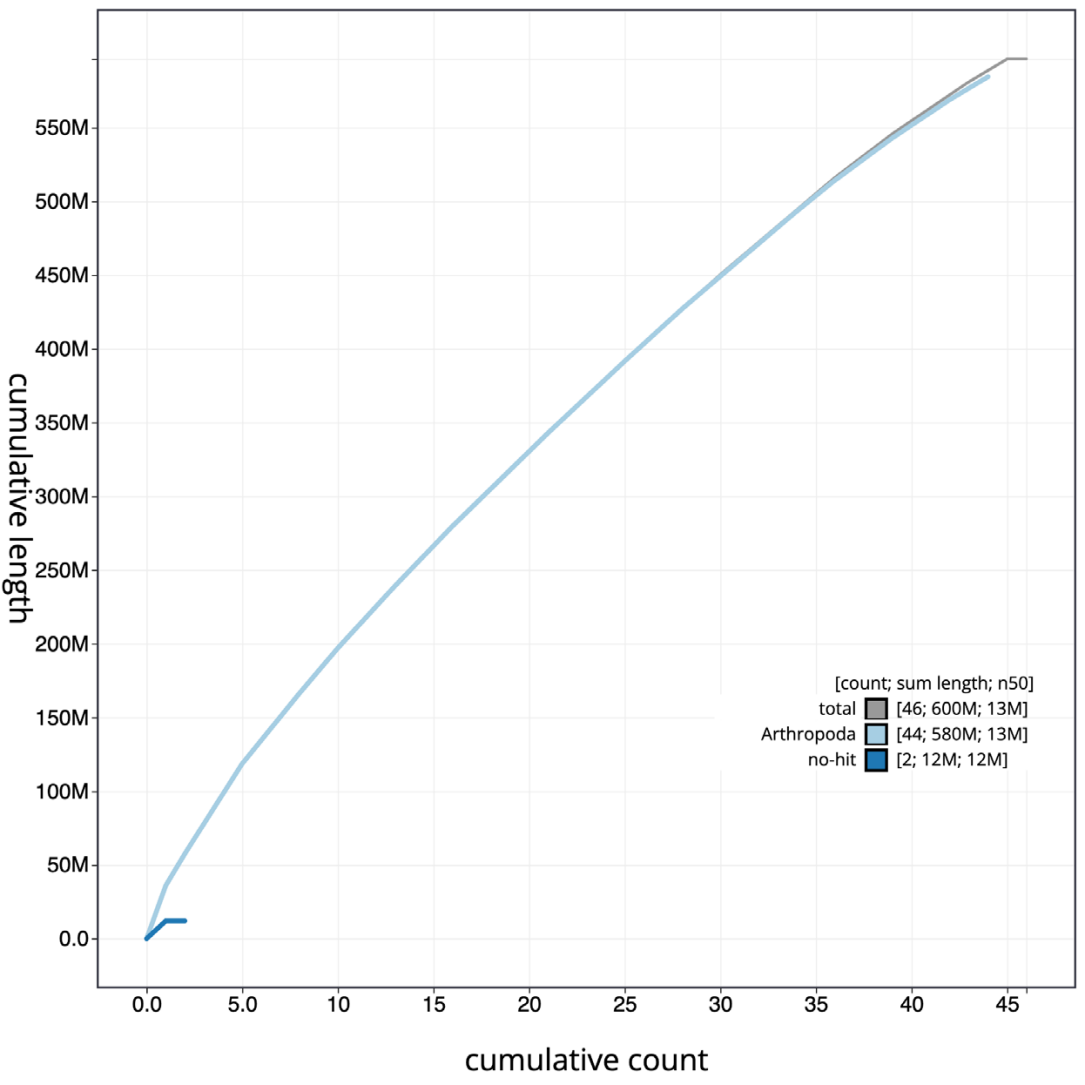
Genome assembly of *Tinea semifulvella*, ilTinSemi1.1: cumulative sequence. BlobToolKit cumulative sequence plot. The grey line shows cumulative length for all scaffolds. Coloured lines show cumulative lengths of scaffolds assigned to each phylum using the buscogenes taxrule. An interactive version of this figure is available at https://blobtoolkit.genomehubs.org/view/ilTinSemi1_1.1/dataset/ilTinSemi1_1.1/cumulative.

**Figure 5.**
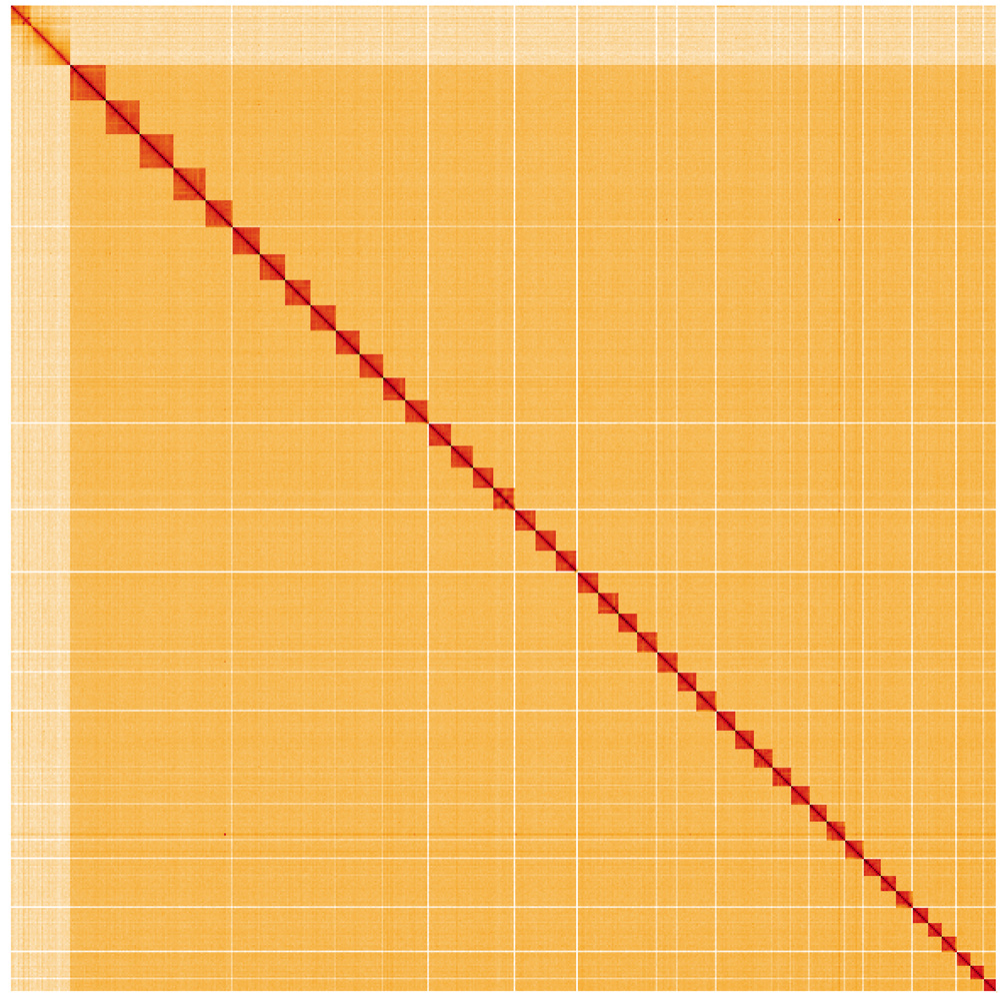
Genome assembly of *Tinea semifulvella*, ilTinSemi1.1: Hi-C contact map. Hi-C contact map of the ilTinSemi1.1 assembly against the specimen ilTinSemi3, visualised using HiGlass. Chromosomes are shown in order of size from left to right and top to bottom. An interactive version of this figure may be viewed at https://genome-note-higlass.tol.sanger.ac.uk/l/?d=HL8wleGpRM2Mr4BHie6J_Q.

**Table 2.** Chromosomal pseudomolecules in the genome assembly of *Tinea semifulvella*, ilTinSemi1.

INSDC accession	Chromosome	Size (Mb)	GC%
OU342585.1	1	21.7	35.4
OU342586.1	2	20.61	35.2
OU342587.1	3	20.57	35.3
OU342588.1	4	19.87	35.2
OU342589.1	5	16.06	35.5
OU342590.1	6	16.01	36.4
OU342591.1	7	15.77	35.5
OU342592.1	8	15.33	35.6
OU342593.1	9	15.2	35.6
OU342594.1	10	14.37	35.8
OU342595.1	11	14.13	35.5
OU342596.1	12	13.82	35.7
OU342597.1	13	13.75	35.9
OU342598.1	14	13.35	35.6
OU342599.1	15	13.3	35.9
OU342600.1	16	12.88	35.8
OU342601.1	17	12.72	35.5
OU342602.1	18	12.62	35.8
OU342603.1	19	12.62	35.9
OU342604.1	20	12.43	35.8
OU342605.1	21	12.37	36.1
OU342606.1	22	12.28	35.9
OU342607.1	23	12.03	35.8
OU342608.1	24	11.97	35.9
OU342609.1	25	11.97	36
OU342610.1	26	11.84	36
OU342611.1	27	11.81	35.9
OU342612.1	28	11.79	36
OU342613.1	29	11.3	36.1
OU342614.1	30	11.09	36.2
OU342615.1	31	11.06	36.4
OU342616.1	32	10.98	36.2
OU342617.1	33	10.94	36
OU342618.1	34	10.93	36.2
OU342619.1	35	10.86	36
OU342620.1	36	10.16	36.4
OU342621.1	37	9.78	36.7
OU342622.1	38	9.7	36.4
OU342623.1	39	9.16	36.2
OU342624.1	40	8.72	36.4
OU342625.1	41	8.69	36.5
OU342626.1	42	8.58	36.6
OU342627.1	43	7.87	36.3
OU342628.1	44	7.64	36.4
OU342584.1	Z	35.94	35
OU342629.1	MT	0.02	19.8

### Genome annotation report

The *T. semifulvella* genome assembly GCA_910589645.1 was annotated using the Ensembl rapid annotation pipeline ( Table 1; https://rapid.ensembl.org/Tinea_semifulvella_GCA_910589645.1/). The resulting annotation includes 20,468 transcribed mRNAs from 11,516 protein-coding and 2,198 non-coding genes.

## Methods

### Sample acquisition and nucleic acid extraction

Three *T. semifulvella* specimens (ilTinSemi1, ilTinSemi2 and ilTinSemi3) were collected using a light trap in Wytham Woods, Oxfordshire (biological vice-county: Berkshire), UK (latitude 51.78, longitude –1.32) on the following dates: 21 September 2019, 13 June 2020 and 5 July 2020, respectively. The specimens were collected and identified by Douglas Boyes (University of Oxford), and snap-frozen on dry ice.

DNA was extracted from whole organism tissue of ilTinSemi1 at the Wellcome Sanger Institute (WSI) Scientific Operations core using the Qiagen MagAttract HMW DNA kit, according to the manufacturer’s instructions.

RNA was extracted from whole organism tissue of ilTinSemi2 in the Tree of Life Laboratory at the WSI using TRIzol, according to the manufacturer’s instructions. RNA was then eluted in 50 μl RNAse-free water and its concentration assessed using a Nanodrop spectrophotometer and Qubit Fluorometer using the Qubit RNA Broad-Range (BR) Assay kit. Analysis of the integrity of the RNA was done using Agilent RNA 6000 Pico Kit and Eukaryotic Total RNA assay.

### Sequencing

Pacific Biosciences HiFi circular consensus and 10X Genomics read cloud DNA sequencing libraries were constructed according to the manufacturers’ instructions. Poly(A) RNA-Seq libraries were constructed using the NEB Ultra II RNA Library Prep kit. DNA and RNA sequencing was performed by the Scientific Operations core at the WSI on Pacific Biosciences SEQUEL II (HiFi), Illumina HiSeq 4000 (RNA-Seq) and HiSeq X Ten (10X) instruments. Hi-C data were also generated from ilTinSemi3 using the Arima v2 kit and sequenced on the Illumina NovaSeq 6000 instrument.

### Genome assembly

Assembly was carried out with Hifiasm ( Cheng *et al.*, 2021) and haplotypic duplication was identified and removed with purge_dups ( Guan *et al.*, 2020). One round of polishing was performed by aligning 10X Genomics read data to the assembly with Long Ranger ALIGN, calling variants with freebayes ( Garrison & Marth, 2012). The assembly was then scaffolded with Hi-C data ( Rao *et al.*, 2014) using SALSA2 ( Ghurye *et al.*, 2019). The assembly was checked for contamination and corrected using the gEVAL system ( Chow *et al.*, 2016) as described previously ( Howe *et al.*, 2021). Manual curation was performed using gEVAL, HiGlass ( Kerpedjiev *et al.*, 2018) and Pretext ( Harry, 2022). The mitochondrial genome was assembled using MitoHiFi ( Uliano-Silva *et al.*, 2022), which performed annotation using MitoFinder ( Allio *et al.*, 2020). The genome was analysed and BUSCO scores generated within the BlobToolKit environment ( Challis *et al.*, 2020). Table 3 contains a list of all software tool versions used, where appropriate.

**Table 3.** Software tools and versions used.

Software tool	Version	Source
BlobToolKit	3.5.2	Challis *et al.*, 2020
freebayes	1.3.1-17-gaa2ace8	Garrison & Marth, 2012
gEVAL	N/A	Chow *et al.*, 2016
Hifiasm	0.12	Cheng *et al.*, 2021
HiGlass	1.11.6	Kerpedjiev *et al.*, 2018
Long Ranger ALIGN	2.2.2	https://support.10xgenomics.com/genome-exome/software/pipelines/latest/advanced/other-pipelines
MitoHiFi	2	Uliano-Silva *et al.*, 2022
PretextView	0.2	Harry, 2022
purge_dups	1.2.3	Guan *et al.*, 2020
SALSA	2.2	Ghurye *et al.*, 2019

### Genome annotation

The Ensembl gene annotation system ( Aken *et al.*, 2016) was used to generate annotation for the *T. semifulvella* genome assembly (GCA_910589645.1). Annotation was created primarily through alignment of transcriptomic data to the genome, with gap filling via protein to-genome alignments of a select set of proteins from UniProt ( UniProt Consortium, 2019).

### Ethics and compliance issues

The materials that have contributed to this genome note have been supplied by a Darwin Tree of Life Partner. The submission of materials by a Darwin Tree of Life Partner is subject to the Darwin Tree of Life Project Sampling Code of Practice. By agreeing with and signing up to the Sampling Code of Practice, the Darwin Tree of Life Partner agrees they will meet the legal and ethical requirements and standards set out within this document in respect of all samples acquired for, and supplied to, the Darwin Tree of Life Project. All efforts are undertaken to minimise the suffering of animals used for sequencing. Each transfer of samples is further undertaken according to a Research Collaboration Agreement or Material Transfer Agreement entered into by the Darwin Tree of Life Partner, Genome Research Limited (operating as the Wellcome Sanger Institute), and in some circumstances other Darwin Tree of Life collaborators.

## Data Availability

European Nucleotide Archive: *Tinea semifulvella* (Fulvous Clothes Moth). Accession number PRJEB45131; https://identifiers.org/ena.embl/PRJEB45131. ( Wellcome Sanger Institute, 2021) The genome sequence is released openly for reuse. The *Tinea semifulvella* genome sequencing initiative is part of the Darwin Tree of Life (DToL) project. All raw sequence data and the assembly have been deposited in INSDC databases. Raw data and assembly accession identifiers are reported in Table 1.

## Author information

Members of the University of Oxford and Wytham Woods Genome Acquisition Lab are listed here: https://doi.org/10.5281/zenodo.4789928.

Members of the Darwin Tree of Life Barcoding collective are listed here: https://doi.org/10.5281/zenodo.4893703.

Members of the Wellcome Sanger Institute Tree of Life programme are listed here: https://doi.org/10.5281/zenodo.4783585.

Members of Wellcome Sanger Institute Scientific Operations: DNA Pipelines collective are listed here: https://doi.org/10.5281/zenodo.4790455.

Members of the Tree of Life Core Informatics collective are listed here: https://doi.org/10.5281/zenodo.5013541.

Members of the Darwin Tree of Life Consortium are listed here: https://doi.org/10.5281/zenodo.4783558.
