# Supplementary material for: The genome sequence of the Fulvous Clothes Moth, Tinea semifulvella (Haworth, 1828)
Source: Wellcome Open Res. 2023 Feb 24;8:104. [Version 1] doi: 10.12688/wellcomeopenres.19079.1 (PMC11069043; doi:10.12688/wellcomeopenres.19079.1)
Supplement: Peer review history

# Reviewer report: Reviewer response for version 1

**DOI:** 10.21956/wellcomeopenres.21153.r77439

**Published:** 2024-05-08

**Authors:** Kuppusamy Sivasankaran

**Affiliations:** 1 Division of Taxonomy and Biodiversity, Entomology Research Institute, Loyola College, Chennai, Tamil Nadu, India

The genome of the Fulvous Clothes Moth, *Tinea semifulvella *(Haworth, 1828) was sequenced and assembled using appropriate techniques.  In this assembly 45 chromosomal pseudomolecules along with the Z sex chromosome have been conformed.

 Minor comment

 In the background of the manuscript first line the genus name ( **T** *inea semifulvella*) first letter is not in italics. The first letter can be changed as italics.

 Above all, I confirm that the manuscript meets the necessary scientific standard and is suitable for indexing.

Are sufficient details of methods and materials provided to allow replication by others?

Yes

Is the rationale for creating the dataset(s) clearly described?

Yes

Are the datasets clearly presented in a useable and accessible format?

Yes

Are the protocols appropriate and is the work technically sound?

Yes

Reviewer Expertise:

Phylogenetic analysis of moths

I confirm that I have read this submission and believe that I have an appropriate level of expertise to confirm that it is of an acceptable scientific standard.

# Reviewer report: Reviewer response for version 1

**DOI:** 10.21956/wellcomeopenres.21153.r77434

**Published:** 2024-05-03

**Authors:** Saurav Baral

**Affiliations:** 1 Tata Institute of Fundamental Research, Bengaluru, India

The findings presented in this paper contribute significantly to the expanding repository of Lepidoptera genomes. In addition to presenting a chromosomally resolved genome, the authors have delivered a meticulous annotation, enhancing the overall utility of the amassed dataset. Despite over 500 lepidopteran genomes having been assembled to the chromosomal level, a mere fraction—less than 50—boast comprehensive annotations. The near-complete annotation achieved in this study holds great promise for advancing research in comparative genomics. Such complete genomic dataset aids significantly to the study of genes, gene families and genome evolution and I would like to both thank and congratulate the authors for producing this dataset. With that said, there are still improvements that can be made on how the data is presented in the paper.

 The paper is concise, which is good. But there are sections of the paper that can be improved.

 Issues:

1. The current image of the specimen, Figure 1, does not contain a proper scale. Please add a proper image of the species, with appropriate scale.
2. “The moth is found in a range of habitats and comes to light.” Do you mean the moth is Diurnal? Please use appropriate scientific words and avoid ambiguous statements.
3. “Manual assembly curation corrected 21 missing or mis-joins and removed one haplotypic duplication, reducing the scaffold number by 30.77% and increasing the scaffold N50 by 5.66%.” Please provide the exact metrics for these in addition to the percentages.
4. “The final assembly has a total length of 596.6 Mb in 45 sequence scaffolds with a scaffold N50 of 12715305 Mb”. The N50 is too large.
5. “All of the assembly sequence was assigned to 45 chromosomal-level scaffolds, representing 44 autosomes and the Z sex chromosome.” Please clarify if there is prior information about the number of chromosomes or if closely related species also show similar chromosome number.
6. Please add a table showing comparison between this genome assembly/annotation and assembly/annotation of some related lepidoptera. This would improve both clarity and provide a comparative context towards understanding this analysis.

This project employs standard methodologies, yielding results that align with findings from analogous Lepidopteran species. However, the authors are encouraged to provide more comprehensive details regarding the Annotation pipeline in the Methods section, which currently lacks specificity. The paper also requires modifications in the Background section for clarity and scientific precision. Additionally, incorporating a comparative analysis within the Genome Sequence Report would enrich the contextual understanding of the metrics presented in the paper. While the quality of the work and the results is commendable, their excellence may not be fully apparent without a broader comparative context. With these minor corrections, the paper may be accepted for indexing.

Are sufficient details of methods and materials provided to allow replication by others?

Partly

Is the rationale for creating the dataset(s) clearly described?

Yes

Are the datasets clearly presented in a useable and accessible format?

Partly

Are the protocols appropriate and is the work technically sound?

Yes

Reviewer Expertise:

I study molecular evolution across gene families using sequences extracted from published genomic datasets.

I confirm that I have read this submission and believe that I have an appropriate level of expertise to confirm that it is of an acceptable scientific standard.

# Reviewer report: Reviewer response for version 1

**DOI:** 10.21956/wellcomeopenres.21153.r72257

**Published:** 2024-01-23

**Authors:** Shu-Jun Wei

**Affiliations:** 1 Beijing Academy of Agriculture and Forestry Sciences, Beijing, China

This study reports the chromosome-level genome for the Fulvous Clothes Moth, *Tinea semifulvella*, assembled using PacBio single-molecule HiFi long reads, 10X Genomics, and Hi-C technologies. The assembly includes forty-five chromosomal pseudomolecules and the mitochondrial genome, along with the Z sex chromosome. The genome assembly is of high quality, with a BUSCO completeness score of 95.3%. Here are some minor comments:

1. Is the scaffold N50 value 12715305 Mb? Please verify the value and unit.
2. Could you provide the BUSCO estimation for the annotated sets of protein-coding genes?

Are sufficient details of methods and materials provided to allow replication by others?

Yes

Is the rationale for creating the dataset(s) clearly described?

Yes

Are the datasets clearly presented in a useable and accessible format?

Yes

Are the protocols appropriate and is the work technically sound?

Yes

Reviewer Expertise:

Population genetics, genomics, and pest control.

I confirm that I have read this submission and believe that I have an appropriate level of expertise to confirm that it is of an acceptable scientific standard.
